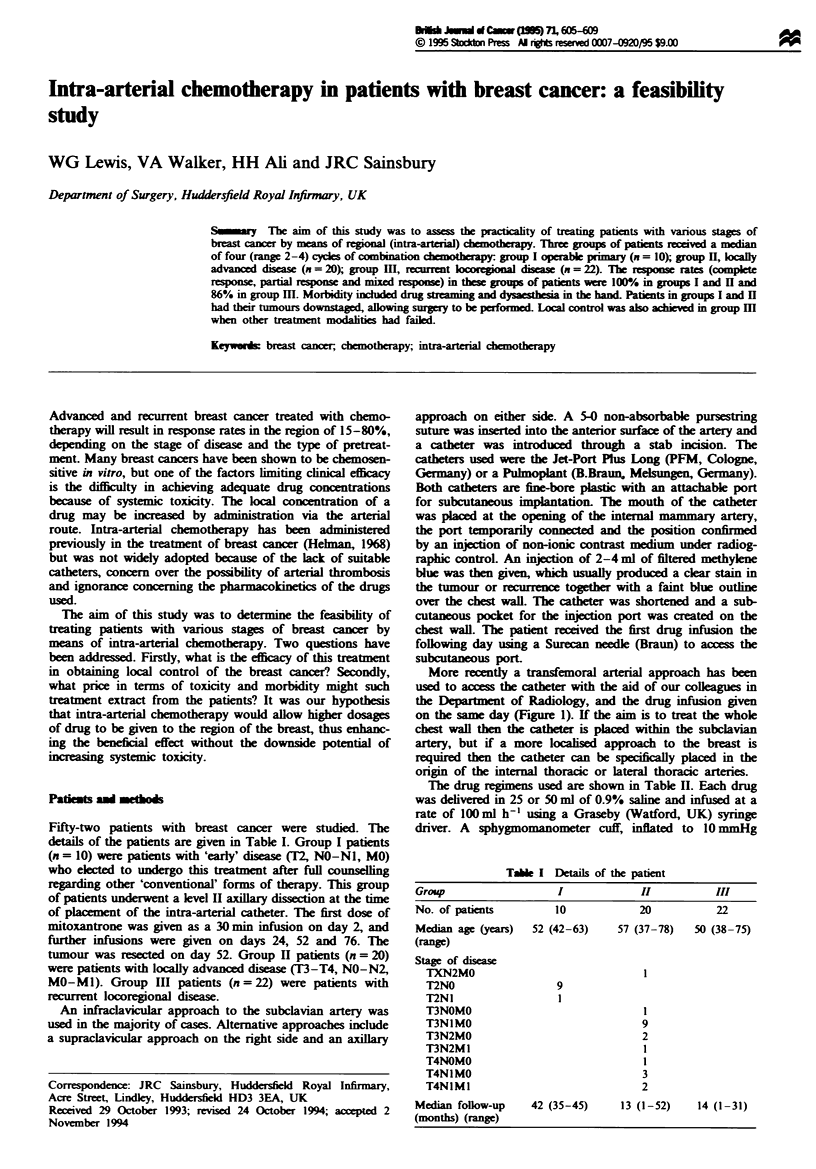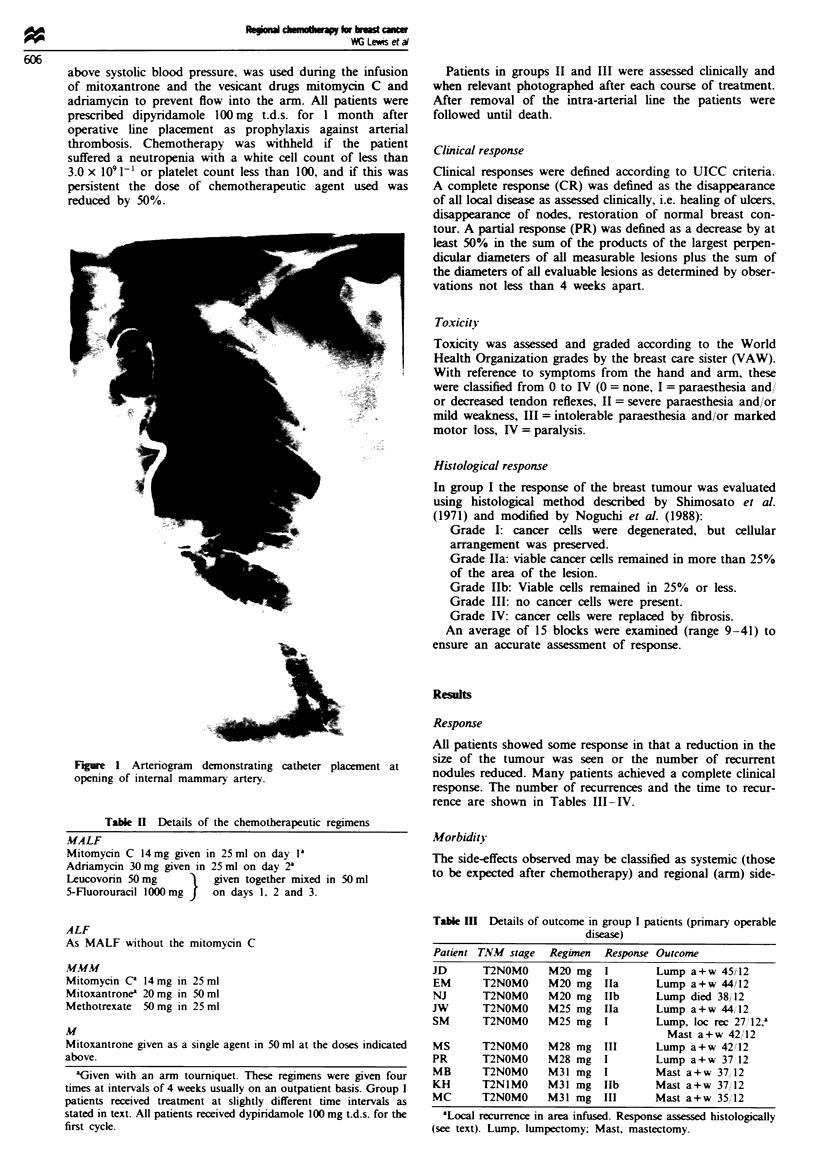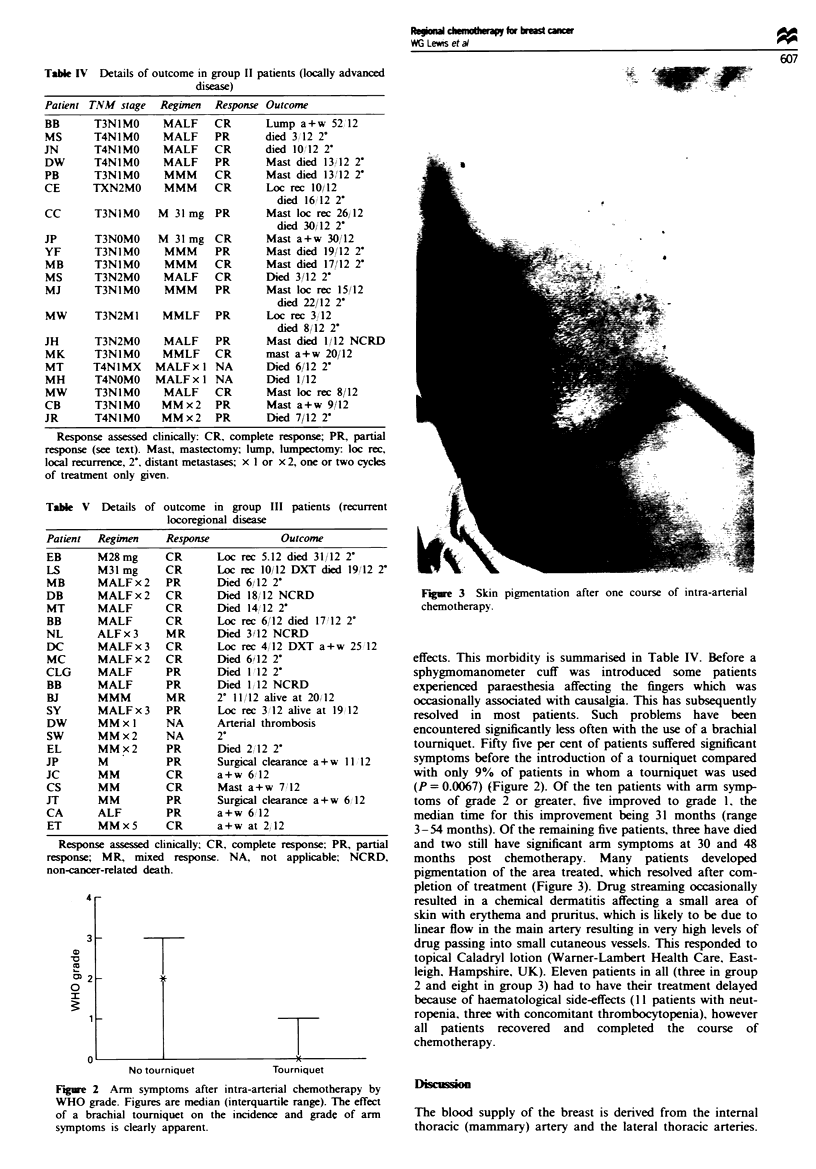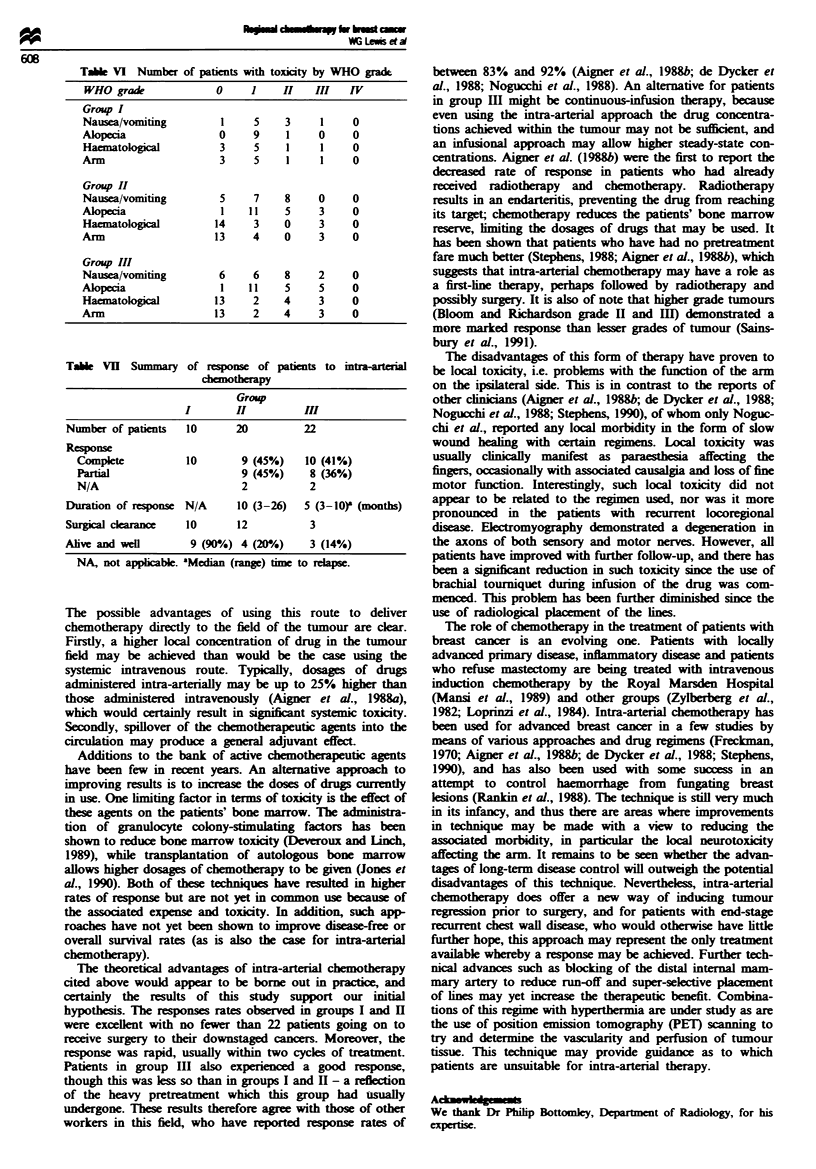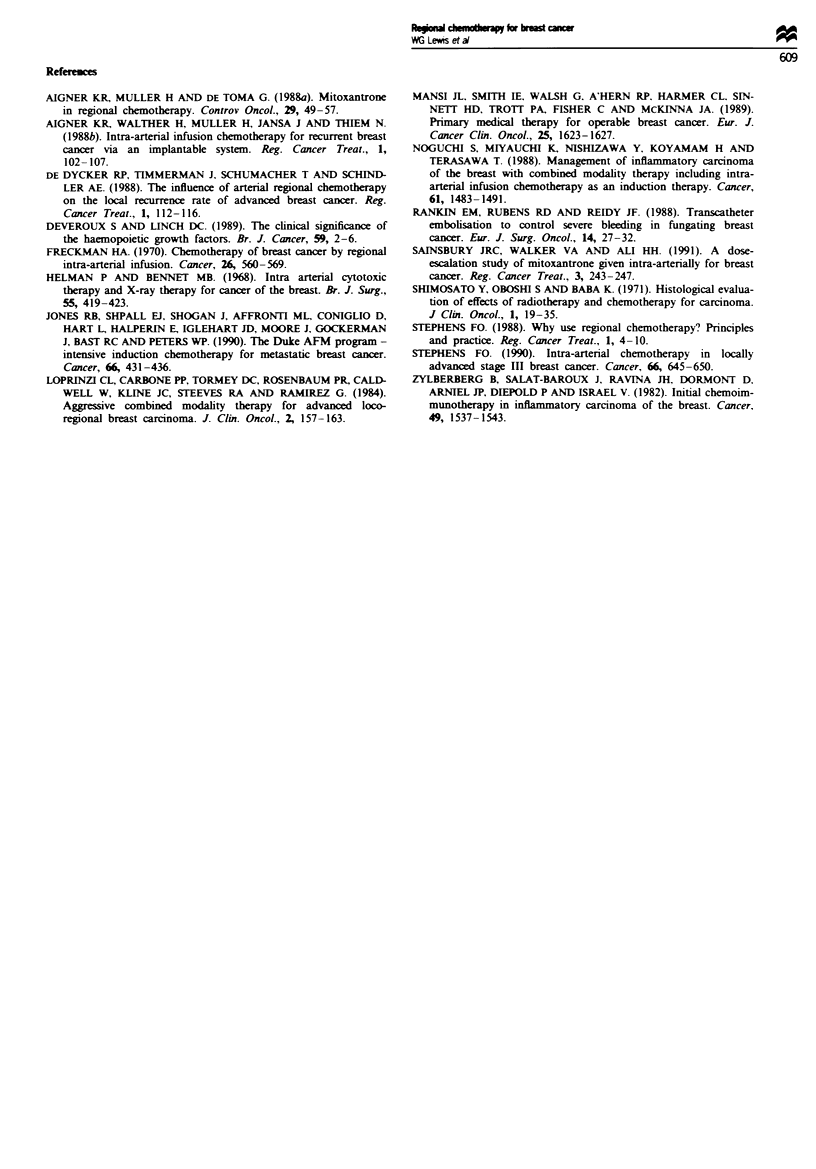# Intra-arterial chemotherapy in patients with breast cancer: a feasibility study.

**DOI:** 10.1038/bjc.1995.117

**Published:** 1995-03

**Authors:** W. G. Lewis, V. A. Walker, H. H. Ali, J. R. Sainsbury

**Affiliations:** Department of Surgery, Huddersfield Royal Infirmary, UK.

## Abstract

**Images:**


© 1995 Stockton Press All rights reserved 0007-0920/95 $9.00

### Intra-arterial chemotherapy in patients with breast cancer: a feasibility study

WG Lewis, VA Walker, HH Ali and JRC Sainsbury

*Department of Surgery, Huddersfield Royal Infirmary, UK*

**Summary** The aim of this study was to assess the practicality of treating patients with various stages of breast cancer by means of regional (intra-arterial) chemotherapy. Three groups of patients received a median of four (range 2-4) cycles of combination chemotherapy: group I operable primary ($n = 10$); group II, locally advanced disease ($n = 20$); group III, recurrent locoregional disease ($n = 22$). The response rates (complete response, partial response and mixed response) in these groups of patients were 100% in groups I and II and 86% in group III. Morbidity included drug streaming and dysaesthesia in the hand. Patients in groups I and II had their tumours downstaged, allowing surgery to be performed. Local control was also achieved in group III when other treatment modalities had failed.

**Keywords:** breast cancer; chemotherapy; intra-arterial chemotherapy

Advanced and recurrent breast cancer treated with chemotherapy will result in response rates in the region of 15-80%, depending on the stage of disease and the type of pretreatment. Many breast cancers have been shown to be chemosensitive *in vitro*, but one of the factors limiting clinical efficacy is the difficulty in achieving adequate drug concentrations because of systemic toxicity. The local concentration of a drug may be increased by administration via the arterial route. Intra-arterial chemotherapy has been administered previously in the treatment of breast cancer (Helman, 1968) but was not widely adopted because of the lack of suitable catheters, concern over the possibility of arterial thrombosis and ignorance concerning the pharmacokinetics of the drugs used.

The aim of this study was to determine the feasibility of treating patients with various stages of breast cancer by means of intra-arterial chemotherapy. Two questions have been addressed. Firstly, what is the efficacy of this treatment in obtaining local control of the breast cancer? Secondly, what price in terms of toxicity and morbidity might such treatment extract from the patients? It was our hypothesis that intra-arterial chemotherapy would allow higher dosages of drug to be given to the region of the breast, thus enhancing the beneficial effect without the downside potential of increasing systemic toxicity.

### Patients and methods

Fifty-two patients with breast cancer were studied. The details of the patients are given in Table I. Group I patients ($n = 10$) were patients with 'early' disease (T2, N0-N1, M0) who elected to undergo this treatment after full counselling regarding other 'conventional' forms of therapy. This group of patients underwent a level II axillary dissection at the time of placement of the intra-arterial catheter. The first dose of mitoxantrone was given as a 30 min infusion on day 2, and further infusions were given on days 24, 52 and 76. The tumour was resected on day 52. Group II patients ($n = 20$) were patients with locally advanced disease (T3-T4, N0-N2, M0-M1). Group III patients ($n = 22$) were patients with recurrent locoregional disease.

An infraclavicular approach to the subclavian artery was used in the majority of cases. Alternative approaches include a supraclavicular approach on the right side and an axillary approach on either side. A 5-0 non-absorbable pursestring suture was inserted into the anterior surface of the artery and a catheter was introduced through a stab incision. The catheters used were the Jet-Port Plus Long (PFM, Cologne, Germany) or a Pulmoplant (B.Braun, Melsungen, Germany). Both catheters are fine-bore plastic with an attachable port for subcutaneous implantation. The mouth of the catheter was placed at the opening of the internal mammary artery, the port temporarily connected and the position confirmed by an injection of non-ionic contrast medium under radiographic control. An injection of 2-4 ml of filtered methylene blue was then given, which usually produced a clear stain in the tumour or recurrence together with a faint blue outline over the chest wall. The catheter was shortened and a subcutaneous pocket for the injection port was created on the chest wall. The patient received the first drug infusion the following day using a Surecan needle (Braun) to access the subcutaneous port.

More recently a transfemoral arterial approach has been used to access the catheter with the aid of our colleagues in the Department of Radiology, and the drug infusion given on the same day (Figure 1). If the aim is to treat the whole chest wall then the catheter is placed within the subclavian artery, but if a more localised approach to the breast is required then the catheter can be specifically placed in the origin of the internal thoracic or lateral thoracic arteries.

The drug regimens used are shown in Table II. Each drug was delivered in 25 or 50 ml of 0.9% saline and infused at a rate of 100 ml $h^{-1}$ using a Graseby (Watford, UK) syringe driver. A sphygmomanometer cuff, inflated to 10 mmHg

**Table I** Details of the patient

| *Group* | *I* | *II* | *III* |
|---|---|---|---|
| No. of patients | 10 | 20 | 22 |
| Median age (years) (range) | 52 (42-63) | 57 (37-78) | 50 (38-75) |
| Stage of disease | | | |
| TXN2M0 | | 1 | |
| T2N0 | 9 | | |
| T2N1 | 1 | | |
| T3N0M0 | | 1 | |
| T3N1M0 | | 9 | |
| T3N2M0 | | 2 | |
| T3N2M1 | | 1 | |
| T4N0M0 | | 1 | |
| T4N1M0 | | 3 | |
| T4N1M1 | | 2 | |
| Median follow-up (months) (range) | 42 (35-45) | 13 (1-52) | 14 (1-31) |

Correspondence: JRC Sainsbury, Huddersfield Royal Infirmary, Acre Street, Lindley, Huddersfield HD3 3EA, UK
Received 29 October 1993; revised 24 October 1994; accepted 2 November 1994

above systolic blood pressure, was used during the infusion of mitoxantrone and the vesicant drugs mitomycin C and adriamycin to prevent flow into the arm. All patients were prescribed dipyridamole 100 mg t.d.s. for 1 month after operative line placement as prophylaxis against arterial thrombosis. Chemotherapy was withheld if the patient suffered a neutropenia with a white cell count of less than $3.0 \times 10^9 l^{-1}$ or platelet count less than 100, and if this was persistent the dose of chemotherapeutic agent used was reduced by 50%.

**Figure 1** Arteriogram demonstrating catheter placement at opening of internal mammary artery.

**Table II** Details of the chemotherapeutic regimens

*MALF*

Mitomycin C 14 mg given in 25 ml on day 1[a]

Adriamycin 30 mg given in 25 ml on day 2[a]

Leucovorin 50 mg, 5-Fluorouracil 1000 mg } given together mixed in 50 ml on days 1, 2 and 3.

*ALF*
As MALF without the mitomycin C

*MMM*

Mitomycin C[a] 14 mg in 25 ml

Mitoxantrone[a] 20 mg in 50 ml

Methotrexate 50 mg in 25 ml

*M*
Mitoxantrone given as a single agent in 50 ml at the doses indicated above.

[a]Given with an arm tourniquet. These regimens were given four times at intervals of 4 weeks usually on an outpatient basis. Group I patients received treatment at slightly different time intervals as stated in text. All patients received dypiridamole 100 mg t.d.s. for the first cycle.

Patients in groups II and III were assessed clinically and when relevant photographed after each course of treatment. After removal of the intra-arterial line the patients were followed until death.

*Clinical response*

Clinical responses were defined according to UICC criteria. A complete response (CR) was defined as the disappearance of all local disease as assessed clinically, i.e. healing of ulcers, disappearance of nodes, restoration of normal breast contour. A partial response (PR) was defined as a decrease by at least 50% in the sum of the products of the largest perpendicular diameters of all measurable lesions plus the sum of the diameters of all evaluable lesions as determined by observations not less than 4 weeks apart.

*Toxicity*

Toxicity was assessed and graded according to the World Health Organization grades by the breast care sister (VAW). With reference to symptoms from the hand and arm, these were classified from 0 to IV (0 = none, I = paraesthesia and/or decreased tendon reflexes, II = severe paraesthesia and/or mild weakness, III = intolerable paraesthesia and/or marked motor loss, IV = paralysis.

*Histological response*

In group I the response of the breast tumour was evaluated using histological method described by Shimosato *et al.* (1971) and modified by Noguchi *et al.* (1988):

Grade I: cancer cells were degenerated, but cellular arrangement was preserved.
Grade IIa: viable cancer cells remained in more than 25% of the area of the lesion.
Grade IIb: Viable cells remained in 25% or less.
Grade III: no cancer cells were present.
Grade IV: cancer cells were replaced by fibrosis.

An average of 15 blocks were examined (range 9–41) to ensure an accurate assessment of response.

### Results

*Response*

All patients showed some response in that a reduction in the size of the tumour was seen or the number of recurrent nodules reduced. Many patients achieved a complete clinical response. The number of recurrences and the time to recurrence are shown in Tables III–IV.

*Morbidity*

The side-effects observed may be classified as systemic (those to be expected after chemotherapy) and regional (arm) side-

**Table III** Details of outcome in group I patients (primary operable disease)

| *Patient* | *TNM stage* | *Regimen* | *Response* | *Outcome* |
|---|---|---|---|---|
| JD | T2N0M0 | M20 mg | I | Lump a+w 45/12 |
| EM | T2N0M0 | M20 mg | IIa | Lump a+w 44/12 |
| NJ | T2N0M0 | M20 mg | IIb | Lump died 38/12 |
| JW | T2N0M0 | M25 mg | IIa | Lump a+w 44/12 |
| SM | T2N0M0 | M25 mg | I | Lump, loc rec 27/12,[a] Mast a+w 42/12 |
| MS | T2N0M0 | M28 mg | III | Lump a+w 42/12 |
| PR | T2N0M0 | M28 mg | I | Lump a+w 37/12 |
| MB | T2N0M0 | M31 mg | I | Mast a+w 37/12 |
| KH | T2N1M0 | M31 mg | IIb | Mast a+w 37/12 |
| MC | T2N0M0 | M31 mg | III | Mast a+w 35/12 |

[a]Local recurrence in area infused. Response assessed histologically (see text). Lump, lumpectomy; Mast, mastectomy.

**Table IV** Details of outcome in group II patients (locally advanced disease)

| *Patient* | *TNM stage* | *Regimen* | *Response* | *Outcome* |
|---|---|---|---|---|
| BB | T3N1M0 | MALF | CR | Lump a+w 52/12 |
| MS | T4N1M0 | MALF | PR | died 3/12 2° |
| JN | T4N1M0 | MALF | CR | died 10/12 2° |
| DW | T4N1M0 | MALF | PR | Mast died 13/12 2° |
| PB | T3N1M0 | MMM | CR | Mast died 13/12 2° |
| CE | TXN2M0 | MMM | CR | Loc rec 10/12 died 16/12 2° |
| CC | T3N1M0 | M 31 mg | PR | Mast loc rec 26/12 died 30/12 2° |
| JP | T3N0M0 | M 31 mg | CR | Mast a+w 30/12 |
| YF | T3N1M0 | MMM | PR | Mast died 19/12 2° |
| MB | T3N1M0 | MMM | CR | Mast died 17/12 2° |
| MS | T3N2M0 | MALF | CR | Died 3/12 2° |
| MJ | T3N1M0 | MMM | PR | Mast loc rec 15/12 died 22/12 2° |
| MW | T3N2M1 | MMLF | PR | Loc rec 3/12 died 8/12 2° |
| JH | T3N2M0 | MALF | PR | Mast died 1/12 NCRD |
| MK | T3N1M0 | MMLF | CR | mast a+w 20/12 |
| MT | T4N1MX | MALF×1 | NA | Died 6/12 2° |
| MH | T4N0M0 | MALF×1 | NA | Died 1/12 |
| MW | T3N1M0 | MALF | CR | Mast loc rec 8/12 |
| CB | T3N1M0 | MM×2 | PR | Mast a+w 9/12 |
| JR | T4N1M0 | MM×2 | PR | Died 7/12 2° |

Response assessed clinically: CR, complete response; PR, partial response (see text). Mast, mastectomy; lump, lumpectomy: loc rec, local recurrence, 2°, distant metastases; × 1 or × 2, one or two cycles of treatment only given.

**Table V** Details of outcome in group III patients (recurrent locoregional disease

| *Patient* | *Regimen* | *Response* | *Outcome* |
|---|---|---|---|
| EB | M28 mg | CR | Loc rec 5.12 died 31/12 2° |
| LS | M31 mg | CR | Loc rec 10/12 DXT died 19/12 2° |
| MB | MALF×2 | PR | Died 6/12 2° |
| DB | MALF×2 | CR | Died 18/12 NCRD |
| MT | MALF | CR | Died 14/12 2° |
| BB | MALF | CR | Loc rec 6/12 died 17/12 2° |
| NL | ALF×3 | MR | Died 3/12 NCRD |
| DC | MALF×3 | CR | Loc rec 4/12 DXT a+w 25/12 |
| MC | MALF×2 | CR | Died 6/12 2° |
| CLG | MALF | PR | Died 1/12 2° |
| BB | MALF | PR | Died 1/12 NCRD |
| BJ | MMM | MR | 2° 11/12 alive at 20/12 |
| SY | MALF×3 | PR | Loc rec 3/12 alive at 19/12 |
| DW | MM×1 | NA | Arterial thrombosis |
| SW | MM×2 | NA | 2° |
| EL | MM×2 | PR | Died 2/12 2° |
| JP | M | PR | Surgical clearance a+w 11/12 |
| JC | MM | CR | a+w 6/12 |
| CS | MM | CR | Mast a+w 7/12 |
| JT | MM | PR | Surgical clearance a+w 6/12 |
| CA | ALF | PR | a+w 6/12 |
| ET | MM×5 | CR | a+w at 2/12 |

Response assessed clinically; CR, complete response; PR, partial response; MR, mixed response. NA, not applicable; NCRD, non-cancer-related death.

**Figure 2** Arm symptoms after intra-arterial chemotherapy by WHO grade. Figures are median (interquartile range). The effect of a brachial tourniquet on the incidence and grade of arm symptoms is clearly apparent.

**Figure 3** Skin pigmentation after one course of intra-arterial chemotherapy.

effects. This morbidity is summarised in Table IV. Before a sphygmomanometer cuff was introduced some patients experienced paraesthesia affecting the fingers which was occasionally associated with causalgia. This has subsequently resolved in most patients. Such problems have been encountered significantly less often with the use of a brachial tourniquet. Fifty five per cent of patients suffered significant symptoms before the introduction of a tourniquet compared with only 9% of patients in whom a tourniquet was used ($P = 0.0067$) (Figure 2). Of the ten patients with arm symptoms of grade 2 or greater, five improved to grade 1, the median time for this improvement being 31 months (range 3–54 months). Of the remaining five patients, three have died and two still have significant arm symptoms at 30 and 48 months post chemotherapy. Many patients developed pigmentation of the area treated, which resolved after completion of treatment (Figure 3). Drug streaming occasionally resulted in a chemical dermatitis affecting a small area of skin with erythema and pruritus, which is likely to be due to linear flow in the main artery resulting in very high levels of drug passing into small cutaneous vessels. This responded to topical Caladryl lotion (Warner-Lambert Health Care, Eastleigh, Hampshire, UK). Eleven patients in all (three in group 2 and eight in group 3) had to have their treatment delayed because of haematological side-effects (11 patients with neutropenia, three with concomitant thrombocytopenia), however all patients recovered and completed the course of chemotherapy.

### Discussion

The blood supply of the breast is derived from the internal thoracic (mammary) artery and the lateral thoracic arteries.

**Table VI** Number of patients with toxicity by WHO grade

| *WHO grade* | *0* | *I* | *II* | *III* | *IV* |
|---|---|---|---|---|---|
| *Group I* | | | | | |
| Nausea/vomiting | 1 | 5 | 3 | 1 | 0 |
| Alopecia | 0 | 9 | 1 | 0 | 0 |
| Haematological | 3 | 5 | 1 | 1 | 0 |
| Arm | 3 | 5 | 1 | 1 | 0 |
| *Group II* | | | | | |
| Nausea/vomiting | 5 | 7 | 8 | 0 | 0 |
| Alopecia | 1 | 11 | 5 | 3 | 0 |
| Haematological | 14 | 3 | 0 | 3 | 0 |
| Arm | 13 | 4 | 0 | 3 | 0 |
| *Group III* | | | | | |
| Nausea/vomiting | 6 | 6 | 8 | 2 | 0 |
| Alopecia | 1 | 11 | 5 | 5 | 0 |
| Haematological | 13 | 2 | 4 | 3 | 0 |
| Arm | 13 | 2 | 4 | 3 | 0 |

**Table VII** Summary of response of patients to intra-arterial chemotherapy

| | | *Group* | |
|---|---|---|---|
| | *I* | *II* | *III* |
| Number of patients | 10 | 20 | 22 |
| Response | | | |
| Complete | 10 | 9 (45%) | 10 (41%) |
| Partial | | 9 (45%) | 8 (36%) |
| N/A | | 2 | 2 |
| Duration of response | N/A | 10 (3–26) | 5 (3–10)[a] (months) |
| Surgical clearance | 10 | 12 | 3 |
| Alive and well | 9 (90%) | 4 (20%) | 3 (14%) |

NA, not applicable. [a]Median (range) time to relapse.

The possible advantages of using this route to deliver chemotherapy directly to the field of the tumour are clear. Firstly, a higher local concentration of drug in the tumour field may be achieved than would be the case using the systemic intravenous route. Typically, dosages of drugs administered intra-arterially may be up to 25% higher than those administered intravenously (Aigner *et al.*, 1988*a*), which would certainly result in significant systemic toxicity. Secondly, spillover of the chemotherapeutic agents into the circulation may produce a general adjuvant effect.

Additions to the bank of active chemotherapeutic agents have been few in recent years. An alternative approach to improving results is to increase the doses of drugs currently in use. One limiting factor in terms of toxicity is the effect of these agents on the patients' bone marrow. The administration of granulocyte colony-stimulating factors has been shown to reduce bone marrow toxicity (Deveroux and Linch, 1989), while transplantation of autologous bone marrow allows higher dosages of chemotherapy to be given (Jones *et al.*, 1990). Both of these techniques have resulted in higher rates of response but are not yet in common use because of the associated expense and toxicity. In addition, such approaches have not yet been shown to improve disease-free or overall survival rates (as is also the case for intra-arterial chemotherapy).

The theoretical advantages of intra-arterial chemotherapy cited above would appear to be borne out in practice, and certainly the results of this study support our initial hypothesis. The responses rates observed in groups I and II were excellent with no fewer than 22 patients going on to receive surgery to their downstaged cancers. Moreover, the response was rapid, usually within two cycles of treatment. Patients in group III also experienced a good response, though this was less so than in groups I and II – a reflection of the heavy pretreatment which this group had usually undergone. These results therefore agree with those of other workers in this field, who have reported response rates of between 83% and 92% (Aigner *et al.*, 1988*b*; de Dycker *et al.*, 1988; Nogucchi *et al.*, 1988). An alternative for patients in group III might be continuous-infusion therapy, because even using the intra-arterial approach the drug concentrations achieved within the tumour may not be sufficient, and an infusional approach may allow higher steady-state concentrations. Aigner *et al.* (1988*b*) were the first to report the decreased rate of response in patients who had already received radiotherapy and chemotherapy. Radiotherapy results in an endarteritis, preventing the drug from reaching its target; chemotherapy reduces the patients' bone marrow reserve, limiting the dosages of drugs that may be used. It has been shown that patients who have had no pretreatment fare much better (Stephens, 1988; Aigner *et al.*, 1988*b*), which suggests that intra-arterial chemotherapy may have a role as a first-line therapy, perhaps followed by radiotherapy and possibly surgery. It is also of note that higher grade tumours (Bloom and Richardson grade II and III) demonstrated a more marked response than lesser grades of tumour (Sainsbury *et al.*, 1991).

The disadvantages of this form of therapy have proven to be local toxicity, i.e. problems with the function of the arm on the ipsilateral side. This is in contrast to the reports of other clinicians (Aigner *et al.*, 1988*b*; de Dycker *et al.*, 1988; Nogucchi *et al.*, 1988; Stephens, 1990), of whom only Nogucchi *et al.*, reported any local morbidity in the form of slow wound healing with certain regimens. Local toxicity was usually clinically manifest as paraesthesia affecting the fingers, occasionally with associated causalgia and loss of fine motor function. Interestingly, such local toxicity did not appear to be related to the regimen used, nor was it more pronounced in the patients with recurrent locoregional disease. Electromyography demonstrated a degeneration in the axons of both sensory and motor nerves. However, all patients have improved with further follow-up, and there has been a significant reduction in such toxicity since the use of brachial tourniquet during infusion of the drug was commenced. This problem has been further diminished since the use of radiological placement of the lines.

The role of chemotherapy in the treatment of patients with breast cancer is an evolving one. Patients with locally advanced primary disease, inflammatory disease and patients who refuse mastectomy are being treated with intravenous induction chemotherapy by the Royal Marsden Hospital (Mansi *et al.*, 1989) and other groups (Zylberberg *et al.*, 1982; Loprinzi *et al.*, 1984). Intra-arterial chemotherapy has been used for advanced breast cancer in a few studies by means of various approaches and drug regimens (Freckman, 1970; Aigner *et al.*, 1988*b*; de Dycker *et al.*, 1988; Stephens, 1990), and has also been used with some success in an attempt to control haemorrhage from fungating breast lesions (Rankin *et al.*, 1988). The technique is still very much in its infancy, and thus there are areas where improvements in technique may be made with a view to reducing the associated morbidity, in particular the local neurotoxicity affecting the arm. It remains to be seen whether the advantages of long-term disease control will outweigh the potential disadvantages of this technique. Nevertheless, intra-arterial chemotherapy does offer a new way of inducing tumour regression prior to surgery, and for patients with end-stage recurrent chest wall disease, who would otherwise have little further hope, this approach may represent the only treatment available whereby a response may be achieved. Further technical advances such as blocking of the distal internal mammary artery to reduce run-off and super-selective placement of lines may yet increase the therapeutic benefit. Combinations of this regime with hyperthermia are under study as are the use of position emission tomography (PET) scanning to try and determine the vascularity and perfusion of tumour tissue. This technique may provide guidance as to which patients are unsuitable for intra-arterial therapy.

**Acknowledgements**

We thank Dr Philip Bottomley, Department of Radiology, for his expertise.

### References

AIGNER KR, MULLER H AND DE TOMA G. (1988a). Mitoxantrone in regional chemotherapy. *Controv Oncol.*, **29**, 49-57.

AIGNER KR, WALTHER H, MULLER H, JANSA J AND THIEM N. (1988b). Intra-arterial infusion chemotherapy for recurrent breast cancer via an implantable system. *Reg. Cancer Treat.*, **1**, 102-107.

DE DYCKER RP, TIMMERMAN J, SCHUMACHER T AND SCHINDLER AE. (1988). The influence of arterial regional chemotherapy on the local recurrence rate of advanced breast cancer. *Reg. Cancer Treat.*, **1**, 112-116.

DEVEROUX S AND LINCH DC. (1989). The clinical significance of the haemopoietic growth factors. *Br. J. Cancer*, **59**, 2-6.

FRECKMAN HA. (1970). Chemotherapy of breast cancer by regional intra-arterial infusion. *Cancer*, **26**, 560-569.

HELMAN P AND BENNET MB. (1968). Intra arterial cytotoxic therapy and X-ray therapy for cancer of the breast. *Br. J. Surg.*, **55**, 419-423.

JONES RB, SHPALL EJ, SHOGAN J, AFFRONTI ML, CONIGLIO D, HART L, HALPERIN E, IGLEHART JD, MOORE J, GOCKERMAN J, BAST RC AND PETERS WP. (1990). The Duke AFM program - intensive induction chemotherapy for metastatic breast cancer. *Cancer*, **66**, 431-436.

LOPRINZI CL, CARBONE PP, TORMEY DC, ROSENBAUM PR, CALDWELL W, KLINE JC, STEEVES RA AND RAMIREZ G. (1984). Aggressive combined modality therapy for advanced locoregional breast carcinoma. *J. Clin. Oncol.*, **2**, 157-163.

MANSI JL, SMITH IE, WALSH G, A'HERN RP, HARMER CL, SINNETT HD, TROTT PA, FISHER C AND MCKINNA JA. (1989). Primary medical therapy for operable breast cancer. *Eur. J. Cancer Clin. Oncol.*, **25**, 1623-1627.

NOGUCHI S, MIYAUCHI K, NISHIZAWA Y, KOYAMAM H AND TERASAWA T. (1988). Management of inflammatory carcinoma of the breast with combined modality therapy including intra-arterial infusion chemotherapy as an induction therapy. *Cancer*, **61**, 1483-1491.

RANKIN EM, RUBENS RD AND REIDY JF. (1988). Transcatheter embolisation to control severe bleeding in fungating breast cancer. *Eur. J. Surg. Oncol.*, **14**, 27-32.

SAINSBURY JRC, WALKER VA AND ALI HH. (1991). A dose-escalation study of mitoxantrone given intra-arterially for breast cancer. *Reg. Cancer Treat.*, **3**, 243-247.

SHIMOSATO Y, OBOSHI S AND BABA K. (1971). Histological evaluation of effects of radiotherapy and chemotherapy for carcinoma. *J Clin. Oncol.*, **1**, 19-35.

STEPHENS FO. (1988). Why use regional chemotherapy? Principles and practice. *Reg. Cancer Treat.*, **1**, 4-10.

STEPHENS FO. (1990). Intra-arterial chemotherapy in locally advanced stage III breast cancer. *Cancer*, **66**, 645-650.

ZYLBERBERG B, SALAT-BAROUX J, RAVINA JH, DORMONT D, ARNIEL JP, DIEPOLD P AND ISRAEL V. (1982). Initial chemoimmunotherapy in inflammatory carcinoma of the breast. *Cancer*, **49**, 1537-1543.